# Water quality in recirculating aquaculture system using woodchip denitrification and slow sand filtration

**DOI:** 10.1007/s11356-020-08196-3

**Published:** 2020-03-10

**Authors:** Petra Lindholm-Lehto, Jani Pulkkinen, Tapio Kiuru, Juha Koskela, Jouni Vielma

**Affiliations:** Aquatic Production Systems, Natural Resources Institute Finland (Luke), Survontie 9A, FI-40500 Jyväskylä, Finland

**Keywords:** Gas chromatography (GC), Heavy metals, Inductively coupled plasma optical emission spectrometry (ICP-OES), Inductively coupled plasma mass chromatography (ICP-MS), Ion chromatography (IC), Rainbow trout, Recirculating aquaculture system (RAS)

## Abstract

In recirculating aquaculture system (RAS), ammonium excreted by the fish is typically transformed to less toxic nitrate by microbial activity in bioreactors. However, nitrate-nitrogen load can be harmful for the receiving water body when released from the RAS facility. A new type of water treatment system for a RAS was designed, including a passive woodchip denitrification followed by a sand filtration introduced into a side-loop of an experimental RAS, rearing rainbow trout (*Oncorhynchus mykiss*). In the process, woodchips acted as a carbon source for the denitrification, aiming at a simultaneous nitrogen removal and reduction of water consumption while sand filtration was used to remove organic matter and recondition the circulating water. A variety of chemical analyses and toxicological tests were performed to study the suitability of the process and to ensure the absence of harmful or toxic substances in the system. The results did not show increased toxicity, and no increased mortality was reported for the raised species. After the start-up of the system, the concentrations of fatty acids (e.g., hexadecanoic acid <LOD-1.21 mg L^−1^) and heavy metals (e.g., Cd < LOD-0.45 μg L^−1^, Pb < LOD-14 μg L^−1^) remained at very low levels and below those of known toxic effects. In the beginning of the experiment, good denitrification efficiency was achieved, but it declined after 1 month, showing the need for improved stability and dimensioning of the application.

**Electronic supplementary material:**

The online version of this article (10.1007/s11356-020-08196-3) contains supplementary material, which is available to authorized users.

## Introduction

Land-based intensive recirculating aquaculture system (RAS) enables reduction in water consumption and nutrient discharge (Dalsgaard et al. 2013), but it often leads to the generation of highly concentrated waste streams, high in solids and nutrients. In a conventional RAS, the external water requirement is adjusted, based on the maximum acceptable concentration of nitrate in the system (Schuster and Stelz 1998; Martins et al. 2010). Typically, about 5% of the system water is replaced daily with clean water to prevent accumulation of nitrate and dissolved organic solids (Masser et al. 1999; Colt 2006; van Rijn et al. 2006). The amount of nitrate must be reduced to avoid toxic effects for the raised species and to further reduce the inlet water consumption. High nitrate concentrations (> 100 mg L^−1^ NO_3_-N, Chen et al. 2002) can be harmful for the raised species but also potentially lead to eutrophication of the receiving waters if released untreated. Eutrophication of water bodies is globally a severe problem (Sharrer et al. 2016). It has been estimated that annual economic losses due to eutrophication is over 2.2 billion US dollars (Dodds et al. 2009).

Denitrification is the process of transforming oxidized nitrogen compounds (nitrite, NO_2_^−^ and nitrate, NO_3_^−^) to reduced elemental gaseous nitrogen (N_2_) via facultative anaerobic microorganisms (van Rijn et al. 2006). In heterotrophic denitrification, bacteria are able to convert nitrate to nitrogen gas under anaerobic conditions, using nitrate as an electron acceptor, a carbon source as the electron donor, and for growth, catalyzed by specific enzymes (Seitzinger et al. 2006; Rivett et al. 2008; Tallec et al. 2008). Denitrification rate depends on several factors, such as temperature, hydraulic retention time (HRT), and microbiology (Christianson et al. 2012). Nitrate removal rate typically increases with increasing temperature, but moderate rates can be achieved even at 1–5 °C (Schipper et al. 2010b). In systems with high nitrogen loadings, rates of nitrogen removal can be limited by temperature or the availability of carbon (Gibert et al. 2008; Schipper et al. 2010b; Warneke et al. 2011). Denitrification increases the alkalinity in the system and returns some of the inorganic carbon lost during nitrification back into the system.

Typically, nitrifying bioreactors are used for the removal of ammonia, transforming it to nitrite and, further to less toxic nitrate to aquatic species. In most recirculating systems, nitrifying filters have been incorporated, but denitrifying filters are still under development. Denitrification has been applied mainly to remove toxic nitrogen compounds and to prevent them from harming the raised species. Additionally, denitrification can be applied to remove nitrate-nitrogen due to increased environmental regulations related to effluent discharge, elimination of organic carbon, and sulfide from the circulating water (van Rijn et al. 2006; von Ahnen et al. 2018). According to some evaluations (Eding et al. 2006), denitrification added to a conventional RAS process decreases the actual production costs per kg of fish due to alkalinity production by denitrification, decreasing the need for external alkalinity, even though it has somewhat higher requirements for electricity and oxygen.

Denitrification is widely used in drinking water (Aslan and Cakici 2007) and wastewater treatment applications (Fernández-Nava et al. 2008). In wastewater treatment with denitrification, commercial electron and carbon donors are often used, such as carbohydrates and organic alcohols. Moreover, agricultural and wood by-products have been tested as a reactor media and denitrification carbon source, such as wheat straw (Aslan and Turkman 2003), and woodchips (Saliling et al. 2007), but some of them offer only limited availability of dissolved organic carbon for the denitrification, leading to low nitrate removal (Robertson et al. 2005). Among natural organic materials, woodchips are the most commonly used in field-scale denitrification due to their good availability, low cost, and good hydraulic permeability (Schipper et al. 2010a). As the water flows through the woodchip bioreactor, oxygen is removed due to bacterial metabolism leading to anoxic environment (Greenan et al. 2006; Warneke et al. 2011). Wood has a high C/N ratio and it can act as source of labile carbon, suitable for long-term denitrification (Gibert et al. 2008; Schipper et al. 2010b). In recent years, woodchip-based denitrification has been applied in RAS in the USA (Lepine et al. 2018) and in Denmark (von Ahnen et al. 2018, 2019), including full-scale applications.

Denitrification in a woodchip bioreactor can range from 2 to 22 g of removed N m^−3^ d^−1^, depending on the type of wastewater (Schipper et al. 2010b; Christianson et al. 2012). Greenan et al. (2006) reported that woodchip bioreactors achieved denitrification of 19–26 g N m^−3^ d^−1^ with 10–80 mg L^−1^ NO_3_-N load. Saliling et al. (2007) achieved denitrification rates of 1360 g N m^−3^ d^−1^ for woodchips with 200 mg NO_3_-N L^−^ but used methanol addition to ensure full availability of dissolved carbon. Saliling et al. (2007) also showed that woodchips are suitable as a reactor media but estimated that their expected life span was only up to 1 year. Later, a life span of over 10 years has been estimated for similar purposes (Sharrer et al. 2016), while 5–15 years has also been reported due to slow degradation of woodchips under anoxic conditions (Schipper et al. 2010a).

Woodchips can contain various compounds toxic to raised species, including salmonids, such as resin acids (Oikari et al. 1983), retene (7-isopropyl-1-methylphenantrene) (Billiard et al. 1999; Oikari et al. 2002), or heavy metals, depending on the wood species (Świetlik et al. 2012) and its place of growth. Long-chained unsaturated fatty acids, such as oleic, linoleic, linolenic, and palmitoleic can contribute to the toxicity of these waste streams (Leach and Thakore 1978). Additionally, organic compounds and nutrients can leach during the start-up of the process, which is one of the downsides of woodchip denitrification bioreactors (Cameron and Schipper 2010; Healy et al. 2012).

Infiltration of water through sand-containing soil removes dissolved and particulate matter from water and improves its quality. In the formation of natural groundwater, retention of dissolved organic compounds into the soil proceeds via physical and chemical retention mechanisms and biological degradation (Wu et al. 2010; Lindroos et al. 2016). Similarly, infiltration of water through the sandy soil layer is also used in artificial recharge of groundwater (ARG, Peters 1998) and widely used in the production of drinking water in the Nordic countries (Kolehmainen et al. 2008). In the application of this study, the circulating water returning from the woodchip bioreactor and denitrification was led into a sand filter to recondition the discharge water before returning it back into circulation.

Denitrification in RAS is still a less studied process, especially without a commercial carbon source and, excluding only a few studies (von Ahnen et al. 2018), often limited to small-scale trials. The aim of this study was to utilize passive water treatment application for denitrification and to reduce water consumption in RAS rearing rainbow trout *Oncorhynchus mykiss*. Additionally, the goal was to identify and quantify organic and inorganic compounds released during the start-up of the system and later during the experiment, to confirm the suitability of the application for water treatment in a RAS and for the raised species.

## Materials and methods

### Experimental setup

Two different-sized passive water treatment systems were connected to randomly selected, individual RAS using randomly allocated duplicate systems per treatment. Additionally, control systems without a passive treatment side-loop were operated accordingly (Table 1). Excluding the side-loop, the regular water treatment units were similar in all RASs. A more detailed description of the experimental RAS facility is reported by Pulkkinen et al. (2018). In brief, each RAS consisted of a 500-L fish tank and a separate water treatment system with total water volume of 1000 L. Solids removal system included a waste feed collector and swirl separator. In the present trial, an up-flow fixed-bed bioreactor (150 L) followed by a moving-bed bioreactor (150 L) were used, filled with 70 L of RK BioElements heavy (750 m^2^ m^−3^) carrier material, stabilized to full maturity prior to the start of the trial. Dissolved carbon dioxide was removed from the water by a forced-ventilated cascade aeration column, with Bio-Blok 200 (EXPO-NET Danmark A/S, Denmark) filter media.

**Table 1:** Operational design of RAS units (*n* = 6): small side-loops, large side-loops, controls, and rearing conditions of rainbow trout (*Oncorhynchus mykiss*) in the experiment

Characteristics	Value	Unit
Water renewal: small side-loop, large side-loop, control	250 100 500	L kg feed^−1^ d^−1^
Small side-loop, large side-loop	25 40	L d^−1^
Fish quantity per tank	300–274	pcs
Fish density: Initial-final	8.0–24.5	kg m^3^
Average fish weight	13.2–43.7	g
Feed quantity	0.1	kg d^−1^
Feed pellet size	1.7–2.5	mm

**Fig. 1:**
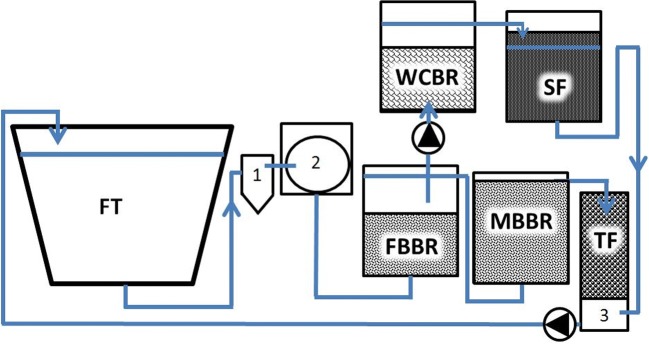
A flowchart of the experimental setup, showing a fish tank (FT), swirl separator, drum filter, fixed-bed reactor (FBBR), moving bed reactor (MBBR), trickling filter (TF), and a side-loop with a woodchip bioreactor (WCBR), and a sand filter (SF)

A side-loop of passive water treatment included a woodchip bioreactor filled with 57-L (small side-loop) or 91-L (large side-loop) unbarked silver birch (*Betula pendula*) woodchips (< 5 cm, effective porosity n_e_ 0.65), aiming for 95% denitrification efficiency (1.4 g NO_3_-N d^−1^ or 2.3 g NO_3_–N d^−1^), with a 1.5-day EBCT (empty bed contact time, hydraulic retention time of the reactor without the woodchips). A sand filter with an effective porosity (n_e_) of 0.35 was packed with 31 cm (90 L, small side-loop) or 50 cm of sand (140 L, large side-loop) with 80% saturation zone and an EBCT of 3.5 days before returning the water back to the pump sump. The amount of water led to the side-loop was measured and adjusted by a peristaltic pump. The water flowed passively first through the woodchip bed and then through the sand filter, exiting the reactor via an overflow (Fig. 1). Denitrification efficiencies were calculated after 3, 6, and 9 weeks of experiment by measuring NO_3_-N by a spectrophotometer (Procedure 8038 Nessler, LCK340, DS 3900, Hach, Loveland, USA).

Surface water from an oligotrophic Lake Peurunka (area of 694 ha, 59,613 m^3^) was used as the clean replacement water at the relative water renewal rate of 25 L day^−1^ (250 L kg^−1^ feed d^−1^, small side-loop), 10 L day^−1^ (100 L kg^−1^ feed d^−1^, large side-loop), and 50 L day^−1^ (500 L kg^−1^ feed d^−1^) for the control systems.

Oxygen levels in the fish tanks were kept at 7.6–8.2 mg L^−1^ by injecting oxygen into the pump sumps. Water temperature was maintained at 15.5 ± 0.7 °C and the pH at 7.2 in the pump sump throughout the experiment. Adjustment of pH was performed by adding 10% NaOH (aq) solution. All measured values were monitored constantly and adjusted when required. Total suspended solids, total organic carbon and turbidity (spectro::lyser, s::can, Vienna, Austria), O_2_ (OxyGuard, Farum, Denmark), CO_2_ (Franatech, Lüneburg, Germany), and pH (ProMinent, Heidelberg, Germany) were measured online every 6 min from the fish tank. Additionally, total ammonia, nitrite, and nitrate nitrogen were monitored weekly by quick laboratory tests (Procedure 8038 Nessler, LCK340, LCK341, UN3316 9 II, Supplementary Table S1), alkalinity by a standard titration method (ISO 9963−1:1994, TitraLab AT1000, Hach, Loveland, USA), and turbidity with a Hach 2100Q Turbidimeter, USA. Circulating water flow rate was set to 0.2 L s^−1^.

### Fish and feeding

The experiment was conducted in the summer of 2018 for 10 weeks. In the beginning, there were 300 fish in each tank, weighing on average 13.2 ± 0.2 g (8.0 kg m^−3^) and, increasing in weight to 43.7 ± 1.0 g (24.5 kg m^−3^) during the experiment. First, the fish were fed with Raisioaqua Circuit Red (Finland) 1.7 mm, and after 4 weeks with 2.5-mm pellets. The main ingredients of feed consisted of fish meal made of Baltic herring and sprat, soya meal, and horse bean, including 0.95–1.15% P and 7.52–7.84% N (Raisioaqua). During the experiment, feed was constantly added 0.1 kg day^−1^ to keep the input of nutrients into the systems constant (Table 1). However, there was an unintended 10% increase in the feeding rate at the experimental week 6, after which it was returned back to 0.1 kg day^−1^. On week 4, an antibiotic orimycin was added (administered in feed) into the system for 10 days to treat the fish against an infection caused by *Flavobacterium psychrophilum*. There was an intermediate weighing after 5 weeks to adjust feeding according to the correct tank biomass. Feed was provided 12 times per day and light for 24 h per day. The fish were visually inspected on a daily basis, and any mortalities were removed and recorded.

### Sample collection

Circulating water was collected from the fish tank once a week from the side-loop after the woodchip bioreactor, and after the sand filter. For the chemical analyses, water samples were collected in 250-mL high-density polyethylene (HDPE) plastic jars with HDPE plastic caps and stored at − 22 °C. For the elemental analyses, the samples were stored at + 2 °C.

### Inorganic anions

#### Instrumentation and chemical analysis

Prior to analysis, water samples were purified by running through a solid-phase extraction (SPE) cartridge (Phenomenex Strata® C18-E, 500 mg/3 mL, 55 μm, 70 Å). The pretreated samples were further filtered through a 0.2-μm syringe filter (13 mm Ø, cellulose, Teknokroma) to avoid blockages in the analysis.

All analyses were conducted on a Dionex DX-500 ion chromatography equipment (Dionex, Sunnyvale, CA, USA), consisting of a gradient pump (AG 40), an anion pre-column (Ion Pac™ AG11-HC-4 μm, 4 mm × 25 mm), an anion separation column (Ion Pac™ AS11-HC-4 μm, 4 mm × 250 mm), anion self-generating suppressor (ASRS 600, 4 mm), an eluent generator (EG40), a conductivity detector (CD20), and an autosampler (AS50).

Elution was performed with a linear gradient from 14 mM KOH for 5 min to 60 mM KOH over the course of 12 min. After 4 min at 60 mM, concentration was decreased to 14 mM, taking 26 min in total. Eluent flow rate was 1.0 mL min^−1^ with the inlet pressure at about 2000 psi, column temperature 30 °C, and sample injection volume 25 μL. Detection was performed with a suppressed conductivity detector and a suppressor current at 149 mA.

#### Method validation

Sodium chloride (NaCl), sodium nitrite (NaNO_2_), sodium nitrate (NaNO_3_), sodium sulfate (Na_2_SO4), and disodium hydrogen phosphate (Na_2_HPO_4_) were used to prepare standard solutions (≥ 99%, Merck). Stock solutions of 5 mg L^−1^ or 10 mg L^−1^ (Na_2_SO_4_) were prepared by diluting an accurate amount of pure standard in UHQ water (internal resistance ≥ 18.2 Ω at 25 °C) by Millipore (Bedford, MA, USA) and filtered through a 0.2-μm syringe filter.

##### LOD, LOQ, linearity

Limit of detection (LOD) and limit of quantification (LOQ) were based on signal-to-noise (S/N) ratio of 3 and 10, respectively. The results were reported as injected to the detector (Supplementary Table S2). LODs ranged between 0.09–1.04 mg L^−1^, and LOQs 0.10–1.15 mg L^−1^.

Linearity of the method was evaluated separately for each compound by plotting the concentrations of five standard solutions against their peak areas. Concentrations ranged between 1 and 100 mg L^−1^. Linear regression analysis was conducted for each standard curve at the confidence interval of 95% (Supplementary Table S2). The regression coefficients were all close to 1, indicating a good linearity. The equations of linearity analysis were used for the quantification of sample concentrations.

##### Precision

Precision of the method was evaluated by performing repeated analyses on following days. A sample spiked with standard solution was analyzed five times during 5 days. Based on the results, the interday and intraday repeatability and precision were calculated (Supplementary Table S3). An analysis of variance (ANOVA) was performed, and the precisions were calculated according to Eqs. 1 and 2, σ_r_ being the residual error, σ_A_ day to day error, and $\overline{x}$ mean of response. The results were expressed as relative standard deviation (RSD, %) according to Destandau et al. (2005) and showed good (< 1%) or intermediate (1–5%) degree of repeatability.

1 $$ {RSD}_{intraday}\left(\%\right)=\frac{\sigma_r}{\overline{x}}100 $$

2 $$ {RSD}_{interday}\left(\%\right)=\frac{\sigma_A}{\overline{x}}100 $$

##### Accuracy

The accuracy of the method was evaluated by comparing the results of introduced standard solutions and calculated results based on the equations of linearity analysis. The results of introduced and calculated concentrations were in good agreement with an error of less than 5% (Supplementary Table S4). Only for nitrate, once the error of recovery was more than 5% (5.2%).

### Fatty acids

#### Chemicals and standards

HPLC grade (≥ 99,8%) methanol, *n*-hexane, pyridine, methyl *tert*-butyl ether (MTBE), and KOH granules (max. 0.002% Na) were purchased from Merck (Darmstadt, Germany), while 25% *o*-bis-(trimethylsilyl)-trifluoroacetamide (BSTFA) with 1% trimethyl chlorosilane (TMCS) from Alfa Aesar (Heysham, Lancashire, UK). Heneicosanoic acid (C_21_H_42_O_2_, purity ≥ 99%, Merck, Saint Louis, MO, USA) was used as an internal standard. Stock solution of 1 mg mL^−1^ was prepared by dissolving an accurate amount of pure standard in MTBE and stored at +2 °C.

#### Sample preparation and analysis

First, the pH of the samples was adjusted to below pH 3 with a few drops of 1 M HCl (aq) to ensure the acidic form of fatty acids if present. For the GC-FID analysis, 4 mL of sample was measured in a screw-capped Kimax tube for liquid-liquid extraction (LLE). 2 mL of MTBE was added, stirred thoroughly, centrifuged at 300 g for 5 min (Centrifuge 1.0), and the clear MTBE layer (supernatant) was collected. A volume of 30 μL of internal standard, heneicosanoic acid (95 μg mL^−1^ in MTBE), was added. The samples were prepared as triplicates and the extraction procedure was repeated three times. Finally, the extracts were evaporated to dryness under a gentle stream of nitrogen. The extracted compounds were derivatized to trimethylsilyl esters. For the derivatization, 760 μL of pyridine (dried with KOH granules) and 330 μL of BSTFA+TMCS were added to the evaporation residue. The solution was heated in an oven at 70 °C for 1 h.

The sample was analyzed with a GC-FID instrument (Shimadzu GC-2010/FID), equipped with a ZB-5MSi column (7HG-G018–11, 30 m × 0.25 mm × 0.25 μm), and an autosampler (AOC-20i). The oven temperature was held at 70 °C for 1 min to equilibrate, heated to 250 °C over the course of 10 min, heated to 300 °C in 5 min, and held for another 5 min. The FID was operated at 300 °C with a sampling rate of 40 msec, helium flow 40 mL min^−1^, and air flow 400 mL min^−1^. All injections were made in the splitless mode, injecting 1 μL of sample.

The compounds were identified by using an Agilent 6890 series/5973 N GC/MSD (Palo Alto, CA, USA) system with a mass spectrometric detector under electron ionization (70 eV), and a Phenomenex Zebron ZB-5MSi (Torrance, CA, USA) capillary column (30 m × 0.25 mm × 0.25 μm). The same oven temperature program was used as with the GC-FID equipment. For the identification of chromatogram peaks, the proper interpretation of the mass spectra was used based on the National Institute of Standards and Technology [NIST] mass spectral library.

#### Method validation

##### LOD, LOQ, linearity

LOD and LOQ were calculated for the standard solution based on signal-to-noise (S/N) ratio of 3 and 10, resulting for LOD 0.12 mg L^−1^ and for LOQ 0.16 mg L^−1^.

Linearity of the method was evaluated by plotting five concentrations ranging between 0.3 and 1.1 mg L^−1^ of internal standard solution (heneicosanoic acid) against their peak areas. Linear regression analysis was conducted for the standard curve at the confidence interval of 95%. The regression coefficient was 0.9934, indicating a good linearity. The equation of linearity analysis was used for the quantification of sample concentrations.

##### Accuracy and precision

Intraday and interday precision was calculated for low (0.3 mg L^−1^) and high (1.1 mg L^−1^) concentration levels. All analyses were performed in a sample matrix spiked with the internal standard heneicosanoic acid as previously reported. Intraday precision of low (0.3 mg L^−1^) concentration was 2.7%, and interday precision 2.7%. For the high concentration (1.1 mg L^−1^), 1.4% intraday and 1.2% interday precisions were reached. The results were expressed as relative standard deviation (RSD, %, *n* = 5) and showed intermediate (1–5%) degree of repeatability.

##### Matrix effect

Matrix effects were determined in circulating water according to Eq. 3 (Garcia-Ac et al. 2009), where SW_S_ is the analyte peak in the spiked circulating water, SW_NS_ analyte peak in the non-spiked circulating water, and W the analyte peak in spiked UHQ water. A value of 100% indicates no matrix effect, while over 100% indicates enhancement and below 100% signal suppression due to matrix effects (Garcia-Ac et al. 2009).

3 $$ Matrix,\%=\left(\frac{SW_S-{SW}_{NS}}{W}\right)100 $$

The matrix effect was studied at five concentrations in the range of 0.3–1.1 mg L^−1^. The matrix effect ranged between 98 and 103%, showing minor matrix effect.

### Elemental analyses

#### Sample digestion and ICP-MS analysis

A microwave acidic digestion of the circulating water samples was performed according to US EPA 2007, method 3015. For practical reasons, weight of the sample was reduced by half to 18 mL; 3 mL of HNO_3_ (65%, Fluka) was added and placed into a polytetrafluoroethylene (PTFE) tube. The tubes were capped and heated in a CEM Mars 6 (Hosmed) microwave oven to 170 °C over the course of 10 min and held for another 10 min at 170 °C (US EPA 2007, method 3015). The samples were left to cool down to 30 °C, transferred into a 40-mL flask and brought to volume with UHQ water. Quality assurance of the digestion method was achieved by performing the analysis of spiked samples and method blanks. The samples were gravimetrically prepared in 1% HNO_3_ (*w*/w) prior to inductively coupled plasma mass chromatography (ICP-MS) analysis. Samples were prepared and analyzed in duplicate, their recovery ranging between 94% and 105% for all elements.

Measurements were performed with a quadrupole-based Perkin Elmer NexION® 350 D ICP-MS system with an octapole collision cell and baffled cyclone electrospray ionization (ESI) cooled to +2 °C. The operating conditions and specifications were listed in Table 2. Before use, the ICP-MS was tuned with a 1 μg L^−1^ tuning solution (Perkin Elmer NexION Setup Solution N8145051). A standard solution containing the selected elements (Al, Cd, Co, Cu, Mn, Ni, and Pb) was prepared at concentration 100 μg L^−1^ (1% NHO_3_, *w*/w), while an internal standard solution (Bi, In, Ga, and Ge, 100 μg L^−1^ in 1% NHO_3_, w/w) was used as a reference and added via a mixing T-piece. All solutions were gravimetrically prepared in 1% HNO_3_, w/w).

**Table 2:** Instrumental parameters and measurement conditions for Perkin Elmer NexION 350 D ICP-MS spectrometer

Isotopes monitored	Al^27^, Cd^111^, Cd^112^, Cd^114^, Co^59^, Cu^63^, Cu^65^, Mn^55^, Ni^58^, Ni^60^, Pb^206^, Pb^207^, Pb^208^
Spray chamber	Cyclonic
RF power	1600 W
Plasma gas flow rate	18 L min^−1^
Nebulizer	PFA-ST
Ar nebulizer gas flow rate	0.85–0.9 L min^−1^
Injector	Perkin Elmer 1.8-mm I.D. Sapphire
Injection volume	1.5 mL
Sampling cone	Ni, 1-mm aperture diameter
Skimmer cone	Ni, 0.4-mm aperture diameter
Scan mode	Peak hopping
Dwell time	50 s
Sweeps per reading	24
Integration time	1200 ms
Readings per replicate	3

##### LOD, LOQ, linearity

As previously mentioned, LODs, LOQs, and linearities (R^2^) were determined for the selected elements (Supplementary Table S5). The indicators were calculated with four concentrations of the standard solution: 0.5 μg L^−1^, 1 μg L^−1^, 5 μg L^−1^, and 10 μg L^−1^. For all selected elements, LODs ranged between 0.02–0.27 μg L^−1^ and LOQs between 0.07–0.92 μg L^−1^, except 2.8 μg L^−1^ for aluminum. The regression coefficients (R^2^) were close to 1 for all selected elements, indicating a good linearity. The equations of linearity analysis were used for the quantification of sample concentrations.

#### ICP-OES analysis

Elemental analyses were performed with a Perkin-Elmer (Optima 8300, Norwalk, CT, USA) inductively coupled plasma optical emission spectrometer (ICP-OES) equipped with a glass concentric nebulizer and a cyclonic spray chamber. The plasma was viewed axially for potassium (K), phosphorous (P), and sulfur (S), but radially in the case of calcium (Ca) and magnesium (Mg). The analytical parameters of the instrument were: RF power 1.5 kW, plasma gas flow rate 8 L min^−1^, auxiliary gas flow rate 0.2 l min^−1^, nebulizer gas flow rate 0.6 L min^−1^, rinse time 10–15 s, and sample uptake 1.5 mL min^−1^. All reagents used were of analytical grade. The measurements were performed in 1% HNO_3_. An external calibration was used by preparing 0.5, 1, 10, 30, and 60 mg L^−1^ standard solutions, containing Ca, K, Mg, P, and S. Accepted relative standard deviation of three replicate measurements was less than 20% with an external calibration. Optimal analytical wavelengths for the measurements were (nm): Ca (315.887), K (766.490), Mg (279.077), P (177.50), and S (182.563). The LODs, LOQs, and linearities were listed in Supplementary Table S6, ranging from 0.29 to 2.2 mg L^−1^ (LODs) and from 1.3 to 8.5 mg L^−1^ (LOQs).

### Toxicity

The toxicity tests were conducted in the laboratory of Ecotoxicology and Risk Assessment, Finnish Environmental Institute (SYKE) for water samples collected from the inlet water from Lake Peurunka, systems with small side-loops, large side-loops, and controls, each with two replicates. The samples were taken right after the start-up of the experiment, representing the most concentrated sample type. Control water (UHQ with 23 different vitamins, and micro- and macronutrients) was used for the control test of acute toxicity to study the fitness of the population.

Acute toxicity test for *Daphnia longispina* was modified from standard ISO 6341. It is based on the survival of 24-h old cubs in 10 ml of undiluted sample solution. The tests were performed with five cubs and five repetitions per treatment. Immobile cubs were counted after 24 h, and again after 48 h.

The inhibitory effect of aqueous samples was studied with standard test ISO 11348-3:2007 for luminescent bacteria *Vibrio fischeri*. It is based on the measurement of luminescence in constant conditions since the luminescence decreases in the case of exposure to hazardous substances. Since *Vibrio fischeri* naturally occurs in sea water, the salt content of undiluted samples was adjusted to 2% with NaCl and pH to 6–8.5. The test was performed with 1234–500 Aboatox™ kit (Aboatox, Finland) stored at −20 °C.

## Results and discussion

### Fish and feeding

There were no differences between the control systems and those with the woodchip bioreactor and the sand filter, when comparing the feed conversion ratio, specific growth rate and fish mortality (Table 3). The fish showed no unusual behavior or signs of stress or discomfort. This suggests that the conditions were suitable for the raised species.

**Table 3:** Feed conversion ratio, specific growth rate, and mortality (± SD) during the experiment

Treatment	Feed conversion ratio	Specific growth rate (% day^−1^)			Mortality (%)	
	Week 2–5	Week 6–9	Week 2–5	Week 6–9	Week 2–5	Week 6–9
Control	0.95 ± 0.02	0.97 ± 0.02	2.0 ± 0.0	0.99 ± 0.03	5.7 ± 2.0	3.3 ± 0.2
Small side-loop	1.00 ± 0.01	1.00 ± 0.03	2.0 ± 0.01	0.96 ± 0.01	6.7 ± 1.1	2.1 ± 0.0
Large side-loop	0.93 ± 0.05	0.91 ± 0.01	2.0 ± 0.07	1.01 ± 0.05	3.9 ± 0.0	1.5 ± 0.5

### Denitrification

**Fig. 2:**
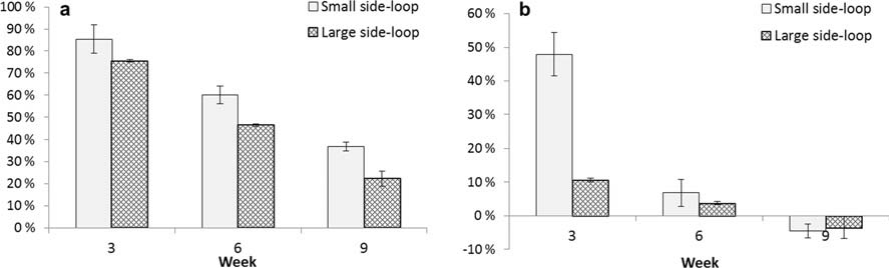
Nitrate removal (%, ± SD, *n* = 24) in the woodchip bioreactor (**a**) and in the sand filter (**b**) after 3, 6, and 9 weeks of the experiment

At the beginning of the experiment, nitrate removal reached 85% in the woodchip bioreactor, and an additional 48% decrease from the remaining NO_3_-N in the sand filter (Fig. 2). This equals the nitrate removal rate of 19.1 g NO_3_-N m^−3^ woodchips d^−1^ in the small side-loop and 16.7 g NO_3_-N m^−3^ d^−1^ in the large side-loop, being in the upper range reported by Schipper et al. (2010b). However, the nitrate-nitrogen removal decreased during the experiment and after 9 weeks only up to 37% efficiencies were reached. The nitrate removal rates decreased from 19.1 g N m^−3^ d^−1^ (week 3) to 15.4 g N m^−3^ d^−1^ (week 6), and 10.0 g N m^−3^ d^−1^ (week 9), respectively, in the small side-loop, while from 16.9 g N m^−3^ d^−1^ (week 3) to 14.1 g N m^−3^ d^−1^ (week 6), and 7.4 g N m^−3^ d^−1^ (week 9) in the large side-loop. The results suggest that the dimensioning of the woodchip bioreactor was insufficient for the nitrogen load. This is supported by the fact that nitrogen removal efficiencies were lower in the large side-loop.

**Fig. 3:**
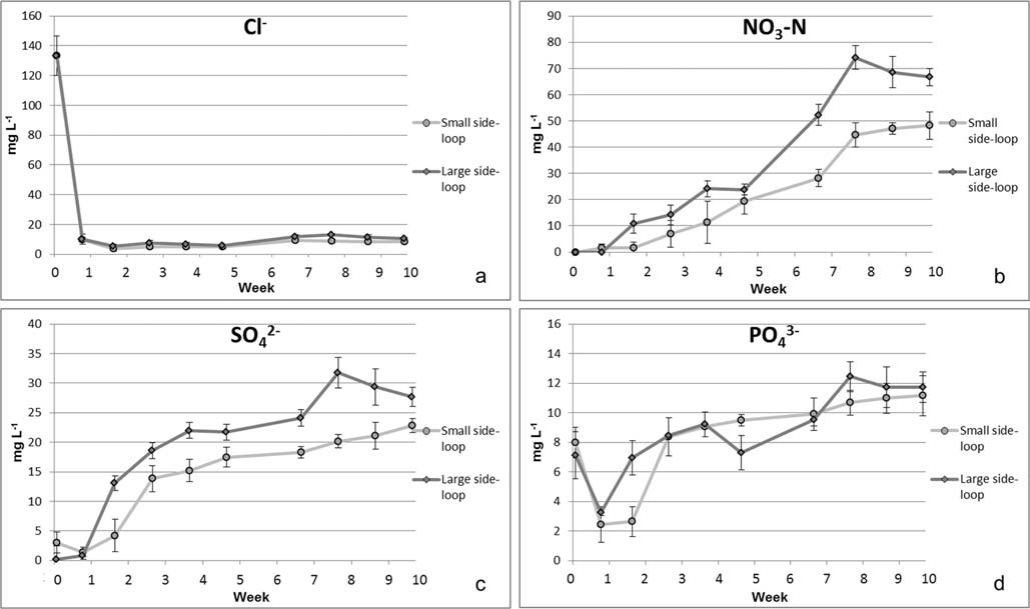
Concentrations of chloride (Cl^−^, a), nitrate-N (NO_3_-N, b), sulfate (SO_4_^2−^, c), and phosphate (PO_4_^3−^, d) (mg L^−1^, ± SD, *n* = 4) in circulating water after the woodchip bioreactor during the 10 weeks of the experiment

Nitrate removal rates of 5.1–21.0 g N m^−3^ d^−1^ have been observed in woodchip bioreactors (Robertson 2010; Hoover et al. 2016; von Ahnen et al. 2016). The removal rate can decrease up to 50% during the first year of operation (Robertson 2010). In this experiment, the removal rates were similar compared to previously reported, however, excluding the rapid decrease in nitrate removal rate. However, there might have been easily dissolving carbon in the woodchips, which induced higher removal rates at the beginning of the experiment. Therefore, the system might have only reached a steady state at experimental week 7, after the nitrate leaching from the bioreactors was leveled (Fig. 3b).

At the experimental week 4, an antibiotic orimycin was added to the system to treat the fish against infection caused by *Flavobacterium psychrophilum* for 10 days. Additionally, there was an unintended 10% increase in the feeding rate at the experimental week 6. The antibiotic addition might have harmed the microbial population in the nitrifying bioreactor, but either is unlikely to cause the decreased efficiencies in the latter part of the experiment. In this study, N_2_O, NO_2_, and NH_4_ were not directly monitored from the outlets of the woodchip bioreactors, leaving the proportions of nitrogen end-products unconfirmed.

### Anions

At the beginning of the experiment, over 140 mg L^−1^ concentrations of chloride were found immediately after starting the experiment (Fig. 3a). Concentrations of readily water-soluble chloride were quickly reduced and remained at about 10 mg L^−1^ level throughout the rest of the experiment. Birch wood contains micronutrients, originating from the soil in the place of growth. Micronutrients, such as chloride, typically occur in wood as cations in aqueous solution (Werkelin et al. 2005). For example, Werkelin et al. (2005) reported 70–110 mg Cl kg^−1^ in dry birch (*Betula pubescens*) wood and 40–330 mg kg^−1^ in birch bark, showing that the woodchips are the most likely source of chloride in the system. Other sources include fish feed and metabolic products of fish (Turcios and Papenbrock 2014).

After the first month of experiment, the nitrate levels, calculated as NO_3_-N, remained below 30 mg L^−1^, but then increased rapidly up to 75 mg L^−1^ (Fig. 3b). The concetrations of nitrate (NO_3_-N) increased in the small side-loop up to 50 mg L^−1^ and in the large side-loop up to 75 mg L^−1^ at the end of the experiment. At first, the concentrations remained lower than those in the tank water but increased up to the same level (Supplementary Table S1). Additionally, denitrification efficiency decreased after 3 weeks of the experiment (Fig. 2), while at that point, concentrations of nitrate in the side-loops started to increase (Fig. 3b).

After the woodchip bioreactor, concentrations of sulfate were first below 20 mg L^−1^ but increased up to 20–35 mg L^−1^ range towards the end of the experiment (Fig. 3c). Levels of phosphate remained more stable throughout the experiment and increased only moderately up to 12 mg L^−1^ in the end of the experiment (Fig. 3d). After the sand filter, the concentrations of sulfates increased above 20 mg L^−1^ and phosphates above 10 mg L^−1^ over the course of the experiment (Supplementary Fig. S1). Concentrations of chloride remained below 15 mg L^−1^, but nitrate (NO_3_-N) increased up to 65 mg L^−1^ at the end of the experiment.

### Fatty acids

**Fig. 4:**
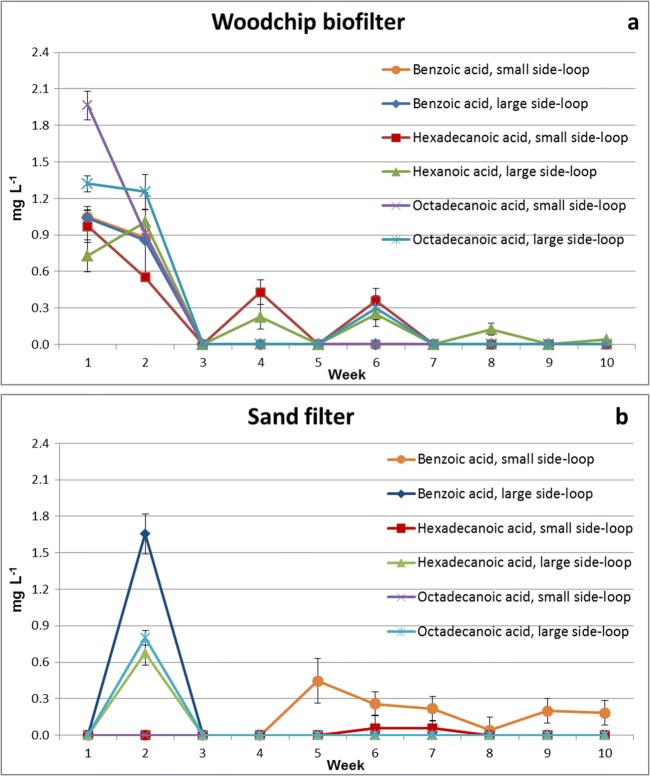
Concentrations (mg L^−1^, ± SD, *n* = 4) of benzoic acid, hexadecanoic acid, and octadecanoic acid in the circulation water, after the woodchip bioreactor (**a**) and the sand filter (**b**), in small and large side-loops during the 10 weeks of the experiment

The long-chained unsaturated fatty acids are known to be toxic to salmonids (Leach and Thakore 1978). Additionally, resin acids originating from softwood are acutely very toxic to fish (Oikari et al. 1983; Peng and Roberts 2000). Therefore, birch woodchips were chosen for the woodchip bioreactor, resulting in no resin acids, or unsaturated fatty acids were found in the circulating water (Fig. 4).

The highest concentrations were found after the start-up of the experiment (Fig. 4), resulting 0.6–2.0 mg L^−1^ after the woodchip bioreactor and 0.7–1.65 mg L^−1^ after the sand filter a few days later. It can be assumed that the fatty acids were first released from the birch woodchips into the circulating water. After 1 week of experiment, the levels of each fatty acid settled at below 0.5 mg L^−1^ throughout the rest of the experiment. Additionally, the concentrations were moderately higher in systems with the large side-loop compared to those with the small side-loop.

Fatty acids have a long carbon chain, giving them their lipophilic and hydrophobic nature. Due to their hydrophobicity, they do not seek into the circulating water, and only low concentrations of fatty acids originating from birch wood were accumulated into the system.

Octanoic acid has biocidal properties, hexanoic acid both biocidal and plant protection properties, while benzoic acid is known to have biocidal properties but also corrosive and hazardous effects to health (ECHA 2019). A low EC50 value (9 mg L^−1^) for benzoic acid has been reported in a chronic study with cyanobacterium *Anabaena inaequalis*, while for the freshwater fish golden ide *Leuciscus idus*, a 48-h LC50 of 460 mg L^−1^ has been determined (WHO 2000). Compared to the known toxicity levels, the concentrations of this study remain below the limit values.

### Elemental analysis

Results of elemental analyses of trace elements have been listed in Table 4. According to EU’s drinking water standard (Council Directive 98/83/EC 1998), limit values have been set for a variety of compounds and elements, while US EPA (2019) has published limit values of certain heavy metals and toxic compounds for aquatic life. It can be used as a reference when evaluating the concentrations found in the circulating water. Especially, the concentrations of lead (Pb) remain below 1 μg L^−1^ (limit value 10 μg L^−1^) for the first 2 weeks of the experiment and then decrease below LOD. In the case of Cd, values were first below 1 μg L^−1^ (limit value 5 μg L^−1^), decreasing to below LOD. Overall, the levels detected in this study were generally well below the limit values, even at the beginning of the experiment. Additionally, the system settles at a low level rapidly after the start-up. This suggests that these trace elements do not seem to accumulate into the system nor pose a risk towards the raised species. This is in agreement with the results of fatty acids (Fig. 4).

**Table 4:** Concentrations of selected trace elements in circulating water during the experiment, after the woodchip bioreactor and after the sand filter (μg L^−1^, ± SD, *n* = 4)

Week	1	2	3	4	5	7	8	9	10
From woodchip bioreactor, small side-loop									
Al, μg L^−1^	<LOD	39 ± 8.1	2.7 ± 1.3	<LOD	<LOD	<LOD	<LOD	<LOD	<LOD
Cd, μg L^−1^	0.32 ± 0.1	0.12 ± 0.1	0.03 ± 0.03	<LOD*	<LOD	<LOD	<LOD	<LOD	<LOD
Co, μg L^−1^	0.5 ± 0.3	0.2 ± 0.1	0.04 ± 0.01	0.2 ± 0.2	<LOD	<LOD	<LOD	<LOD	<LOD
Cu, μg L^−1^	<LOD	11 ± 1.1	2.3 ± 0.4	1.4 ± 0.9	21 ± 6.5	12 ± 11	8.8 ± 6.5	8.2 ± 3.4	4.5 ± 1.5
Mn, μg L^−1^	1100 ± 45	69 ± 6.2	10 ± 1.4	64 ± 1.6	99 ± 0.2	60 ± 14	54 ± 5.4	18 ± 17	55 ± 6.9
Ni, μg L^−1^	<LOD	1.2 ± 0.2	17 ± 14	<LOD	<LOD	<LOD	<LOD	<LOD	<LOD
Pb, μg L^−1^	<LOD	0.9 ± 0.2	<LOD	<LOD	<LOD	<LOD	<LOD	<LOD	<LOD
From woodchip bioreactor, large side-loop									
Al, μg L^−1^	<LOD	55 ± 7.0	1.1 ± 1.1	<LOD	<LOD	<LOD	<LOD	<LOD	<LOD
Cd, μg L^−1^	0.10 ± 0.1	0.45 ± 0.4	0.01 ± 0.01	0.12 ± 0.03	0.18 ± 0.05	<LOD	<LOD	<LOD	<LOD
Co, μg L^−1^	0.3 ± 0.1	0.5 ± 0.6	0.03 ± 0.01	0.2 ± 0.1	<LOD	<LOD	<LOD	<LOD	<LOD
Cu, μg L^−1^	<LOD	16 ± 3.4	2.3 ± 0.1	10 ± 2.2	6.5 ± 3.0	7.2 ± 2.5	7.8 ± 0.6	6.0 ± 1.8	4.7 ± 1.1
Mn, μg L^−1^	1100 ± 180	120 ± 5.4	1.4 ± 1.0	18 ± 14	99 ± 1.5	39 ± 4.9	28 ± 2.0	30 ± 2.5	72 ± 4.9
Ni, μg L^−1^	<LOD	1.2 ± 0.4	63 ± 9.6	<LOD	<LOD	<LOD	<LOD	<LOD	<LOD
Pb, μg L^−1^	<LOD	0.6 ± 0.1	<LOD	<LOD	<LOD	<LOD	<LOD	<LOD	<LOD
From sand filter, small side-loop									
Al, μg L^−1^	3800 ± 970	81 ± 32	2.0 ± 0.5	<LOD	<LOD	<LOD	<LOD	<LOD	<LOD
Cd, μg L^−1^	<LOD	0.15 ± 0.05	0.02 ± 0.01	<LOD	<LOD	<LOD	<LOD	<LOD	<LOD
Co, μg L^−1^	4.8 ± 0.9	0.2 ± 0.1	0.3 ± 0.02	1.3 ± 0.6	1.6 ± 0.9	<LOD	<LOD	0.6 ± 0.2	1.0 ± 0.2
Cu, μg L^−1^	<LOD	12 ± 0.7	3.2 ± 0.6	19 ± 11	21 ± 7.2	12 ± 2.9	16 ± 1.4	15 ± 6.0	11 ± 3.5
Mn, μg L^−1^	330 ± 49	21 ± 8.7	26 ± 3.3	24 ± 4.5	9.3 ± 0.2	91 ± 8.4	160 ± 31	280 ± 6.8	190 ± 1.0
Ni, μg L^−1^	<LOD	1.4 ± 0.2	0.4 ± 0.1	<LOD	<LOD	<LOD	<LOD	<LOD	<LOD
Pb, μg L^−1^	5.6 ± 1.6	0.8 ± 0.3	<LOD	<LOD	<LOD	<LOD	<LOD	<LOD	<LOD
From sand filter, large side-loop									
Al, μg L^−1^	6900 ± 4100	92 ± 16	4.2 ± 2.6	<LOD	<LOD	<LOD	<LOD	<LOD	<LOD
Cd, μg L^−1^	0.10 ± 0.06	0.14 ± 0.01	0.01 ± 0.01	<LOD	<LOD	<LOD	<LOD	<LOD	<LOD
Co, μg L^−1^	9.3 ± 6.7	0.8 ± 0.1	0.1 ± 0.06	0.9 ± 0.1	1.3 ± 0.4	<LOD	1.3 ± 0.9	2.7 ± 1.4	2.3 ± 0.6
Cu, μg L^−1^	24 ± 1.6	35 ± 5.0	5.2 ± 0.6	27 ± 2.4	31 ± 14	21 ± 4.8	27 ± 3.6	24 ± 12	12 ± 2.0
Mn, μg L^−1^	670 ± 14	40 ± 10	4.8 ± 4.4	19 ± 1.4	7.0 ± 0.8	180 ± 22	240 ± 2.2	290 ± 71	390 ± 14
Ni, μg L^−1^	7.9 ± 2.8	4.5 ± 0.7	0.7 ± 0.1	<LOD	<LOD	<LOD	1.2 ± 0.9	0.8 ± 1.0	<LOD
Pb, μg L^−1^	14 ± 12	0.9 ± 0.2	<LOD	<LOD	<LOD	<LOD	<LOD	<LOD	<LOD

^*^LOD Level of detection

Compared to concentrations detected by Martins et al. (2011) in a RAS rearing Nile tilapia *Oreochromis niloticus* at similar water renewal rates, the concentrations were in the same range or lower. Only manganese was found at higher levels in this study. On the other hand, van Bussel et al. (2014) found manganese 422 μ L^−1^ at water renewal rate of 10 L kg^−1^ d^−1^ and 40 μg L^−1^ at 33 L kg^−1^ d^−1^ in a marine RAS rearing juvenile turbot *Psetta maxima*. Additionally, the concentrations remained below the limit values for chronic exposure for aquatic life (Cd 0.72 μg L^−1^, Ni 52 μg L^−1^, Pb 3.2 μg L^−1^) set by the US EPA (US EPA 2019, Table 5).

**Table 5:** Concentrations (μg L^−1^) of acute or chronic toxicity, or limits for optimum water quality for aquatic life

Ca, μg L^−1^	4–160	Hatchery water, trout	Piper et al. 1986
Cd, μg L^−1^	Acute 1.8; chronic 0.72 acute 33; chronic 7.9	In fresh water In salt water	US EPA (2019)*
Cu, μg L^−1^	Acute 4.8; chronic 3.1 50–130	In salt water 96 h LC50 for rainbow trout	US EPA (2019)* Gündoğdu 2008
Fe, μg L^−1^	> 0–150,500	Total, hatchery water, trout Ferric ion	Piper et al. 1986
Mn, μg L^−1^	> 0–10	Hatchery water, trout	Piper et al. 1986
Ni, μg L^−1^	acute 470; chronic 52 acute 74; chronic 8.2	In fresh water In salt water	US EPA (2019)*
P, μg L^−1^	10–3000	Hatchery water, trout	Piper et al. 1986
Pb, μg L^−1^	> 0–30 acute 82; chronic 3.2 acute 140; chronic 5.6	For salmonids In fresh water In salt water	Piper et al. 1986 US EPA (2019)*

*100 mg L^−1^ water hardness as CaCO_3_

In the case of manganese, the concentrations ranged from 330 to 1100 μg L^−1^ in the beginning of the experiment. After the start-up of the system, concentrations decreased even to a few μg L^−1^ of manganese in some cases (Table 4) but increased again to 55–390 μg L^−1^ range in the end of the experiment. Manganese is typically of geological origin and one of the most common heavy metals in soil. In Finland, the average levels of manganese (285 mg kg^−1^, Rasilainen et al. 2007) are lower than on average in the earth crust (630 mg kg^−1^, Kousa et al. 2017). In this study, the sand was collected locally from the area where soil is known to contain manganese. Precipitation of manganese proceeds at pH range 4–7, but it is also affected by the bicarbonate and sulfate concentrations (Kousa et al. 2017). However, the dependence between pH and the amount of release manganese has not been fully resolved (Kousa et al. 2017). An upper limit of 10 μg L^−1^ has been set for optimum trout hatchery water (Piper et al. 1986), but according to our knowledge, limit values for optimum circulating water has not been set.

In the latter part of the experiment, concentrations of manganese increased, as well as those of sulfate (Table 4, Fig. 3c), which may have promoted the release of manganese from the sand filter. Concentration of manganese found in the inlet water from Lake Peurunka was only 6.2 ± 3.3 μg L^−1^, which cannot explain the increased values after the sand filter. This suggests that process conditions of the system have a more substantial effect on the manganese content in the circulating water than its content in the sand filter sand or in the inlet water.

**Fig. 5:**
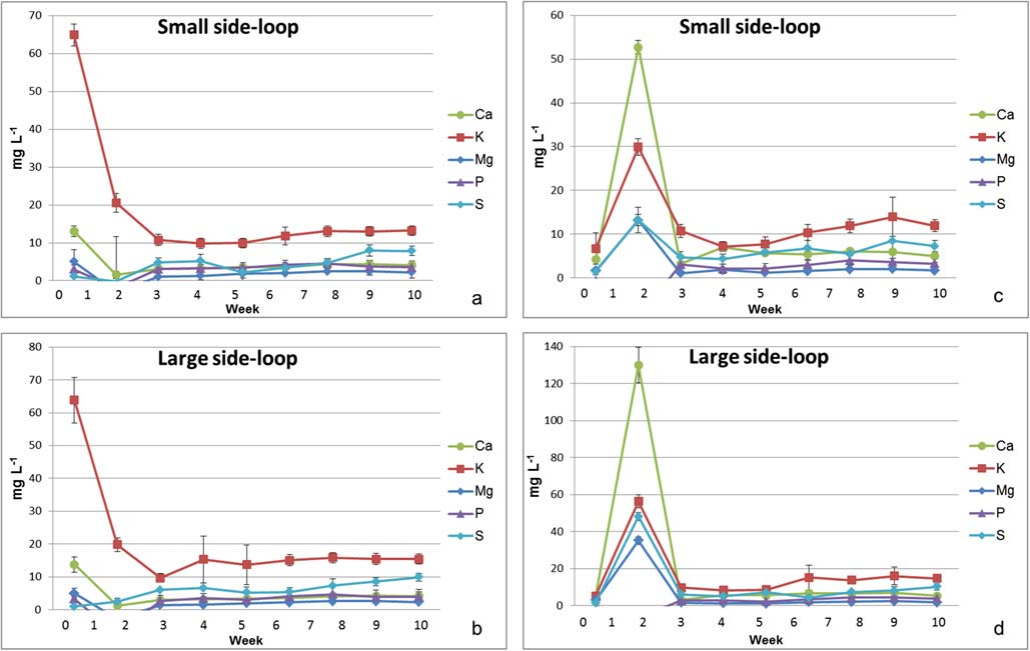
Concentrations of calcium (Ca), potassium (K), magnesium (Mg), phosphorous (P), and sulfur (S) (mg L^−1^ ± SD, *n* = 4) in circulating water after the woodchip bioreactor (**a** small side-loop, **b** large side-loop) and after the sand filter (**c** small side-loop, **d** large side-loop) during the 10 weeks of the experiment

At the beginning of the experiment, increased concentrations up to 70 mg L^−1^ (K) of selected elements were detected (Fig. 5a, b) after the woodchip bioreactor and up to 130 mg L^−1^ (K) after the sand filter (Fig. 5c, d) after 1 week of the experiment. As in the case of most trace elements (Table 4), the concentrations decreased rapidly and remained below 20 mg L^−1^ throughout the rest of the experiment. These elements can be of woodchip origin and thus originate from the woodchip bioreactor. For example, Werkelin et al. (2005) reported concentration ranges 700–900 mg kg^−1^ for Ca, 500–600 mg kg^−1^ for K, 110–160 mg kg^−1^ for Mg, and 50–60 mg kg^−1^ for P in birch (*Betula pubescens*) stem wood, while bark can contain even higher levels (Ca 7100–5500 mg kg^−1^, 2100–2300 mg kg^−1^, P 460–300 mg kg^−1^, Werkelin et al. 2005). Other limit values for acute and chronic toxicity in water and limits for optimum water quality for aquatic life or for salmonids have been listed in Table 5.

### Toxicity

The circulation waters studied did not lead to inhibition of luminescent bacteria *Vibrio fischeri* during the acute exposure. Based on the results, the circulation water did not show inhibitive effects (Supplementary Table S7).

In the acute toxicity test for *Daphnia longispina*, 40% immobility occurred in the control water of the test control. For the circulating water, the rate of mortality (immobility) ranged widely from 0% to 100% but did not show a clear trend between the treatments (Supplementary Table S8). However, the rate of mortality (immobility) was lower in the inlet water (4%) than in the circulating water, except in one of the systems with a small side-loop (0%). The results suggest that circulating water can show toxic effects to some species, but the toxic effects do not seem to be caused by the units of the side-loops.

## Conclusions

This study represents a new process design for circulating water treatment and denitrification in RAS by combining denitrification in a woodchip bioreactor and slow sand filtration. The results show that birch woodchips act as a carbon source and provide surface area for the denitrification. The levels of the anions studied remained at reasonable levels in the rearing tanks and in the side-loops for the first part of the experiment, unlikely causing discomfort or harm for the raised species. However, as the experiment proceeded, the denitrification decreased and nitrate levels increased, suggesting an imbalance of the system and insufficient dimensioning of the reactors. However, this was the first experiment applying the new process configuration of water treatment for RAS. This shows that an improved dimensioning is required to ensure proper function of the woodchip bioreactor and the sand filter. Overall, the concentrations of compounds observed in the system were low, and only in some cases increased concentrations of nitrate, sulfate, and manganese were found. This suggests the suitability of the process for water treatment in RAS, aiming at decreasing the water consumption and growing healthy fish.

This was the first trial of this process design, requiring further studies of the denitrification efficiency and process control in the long run. Additionally, the developed analytical methods to study heavy metals, fatty acids, and anions can be applied to other processes, including large-scale facilities. Later, it is of interest to ensure that N_2_O will not be released and to ensure a good efficiency of denitrification.

## Electronic supplementary material

ESM 1 (DOCX 148 kb)
