# Supplementary material for: Water quality in recirculating aquaculture system using woodchip denitrification and slow sand filtration
Source: Environ Sci Pollut Res Int. 2020 Mar 10;27(14):17314–28. doi: 10.1007/s11356-020-08196-3 (PMC7192871; doi:10.1007/s11356-020-08196-3)
Supplement: Supplementary file 1 — (DOCX 148 kb) [file 11356_2020_8196_MOESM1_ESM.docx]

Supplementary table S1. Weekly measurements of ammonium (HN_3_-N, mg L^-1^), nitrite (NO_2_-N, mg L^-1^), nitrate (NO_3_-N, mg L^-1^), sulfate (SO_4_^2-^, mg L^-1^), alkalinity (mg L^-1^), pH, and turbidity (FNU) in the rearing tank water. Systems with a small side-loop: A1, A2, large side-loop: A6, A7, and controls without a side-loop A3, A8.

| A1 | | | | | | | | |
| --- | --- | --- | --- | --- | --- | --- | --- | --- |
| Week | TAN, mg/L | NH_3_‑N, mg/L | NO_2_-N, mg/L | NO_3_-N, mg/L | SO_4,_ mg/L | Alkalinity, mg/L | pH | Turbidity, FNU |
| 1 | 1.02 | 0,000 | 0.339 | 45.1 |  |  |  |  |
| 2 | 1.07 | 0,011 | 0.245 | 52.6 | 20 | 82.1 | 7.61 | 8.60 |
| 3 | 1.01 | 0,000 | 0.218 | 56.6 | 17 | 87.1 | 7.69 | 7.12 |
| 4 | 0.85 | 0,006 | 0.180 | 54.6 | 20 | 82.1 | 7.34 | 6.53 |
| 5 | 0.98 | 0,005 | 0.173 | 56.4 | 20 | 93.0 | 7.36 | 7.48 |
| 6 | 0.82 | 0,007 | 0.197 | 54.4 | 20 | 76.6 | 7.38 | 3.66 |
| 7 | 0.90 | 0,004 | 0.166 | 65.2 | 24 | 99.5 | 7.28 | 3.91 |
| 8 | 1.14 | 0,005 | 0.193 | 71.6 | 33 | 99.7 | 7.29 | 6.84 |
| 9 | 1.26 | 0,005 | 0.182 | 67.6 | 27 | 82.3 | 7.21 | 9.58 |
| 10 | 1.14 | 0,006 | 0.182 | 69.8 | 27 | 78.6 | 7.25 | 9.45 |

| A2 | | | | | | | | |
| --- | --- | --- | --- | --- | --- | --- | --- | --- |
| Week | TAN, mg/L | NH_3_‑N, mg/L | NO_2_-N, mg/L | NO_3_-N, mg/L | SO_4_, mg/L | Alkalinity, mg/L | pH | Turbidity, FNU |
| 1 | 1.04 | 0,000 | 0.336 | 40.0 |  |  |  |  |
| 2 | 1.17 | 0,008 | 0.279 | 48.0 | 16 | 72.3 | 7.46 | 12.7 |
| 3 | 1.21 | 0,000 | 0.252 | 51.0 | 18 | 91.2 | 7.79 | 11.5 |
| 4 | 1.25 | 0,009 | 0.172 | 53.6 | 20 | 111.1 | 7.44 | 10.4 |
| 5 | 1.35 | 0,005 | 0.191 | 55.0 | 18 | 68.1 | 7.12 | 14.1 |
| 6 | 1.14 | 0,007 | 0.151 | 55.4 | 19 | 57.2 | 7.25 | 7.25 |
| 7 | 0.96 | 0,007 | 0.108 | 67.6 | 25 | 115.6 | 7.36 | 6.41 |
| 8 | 1.12 | 0,008 | 0.129 | 70.4 | 26 | 141.5 | 7.48 | 7.34 |
| 9 | 0.92 | 0,008 | 0.155 | 67.6 | 26 | 134.0 | 7.40 | 7.24 |
| 10 | 0.66 | 0,008 | 0.141 | 65.2 | 24 | 142.8 | 7.50 | 2.84 |

| A3 | | | | | | | | |
| --- | --- | --- | --- | --- | --- | --- | --- | --- |
| Week | TAN, mg/L | NH_3_‑N, mg/L | NO_2_-N, mg/L | NO_3_-N, mg/L | SO_4_, mg/L | Alkalinity, mg/L | pH | Turbidity, FNU |
| 1 | 0.91 | 0,000 | 0.325 | 38.1 |  |  |  |  |
| 2 | 0.90 | 0,006 | 0.239 | 46.1 | 16 | 68.0 | 7.36 | 7.32 |
| 3 | 0.95 | 0,000 | 0.187 | 45.4 | 14 | 65.6 | 7.63 | 9.18 |
| 4 | 0.94 | 0,004 | 0.162 | 58.6 | 17 | 63.1 | 7.22 | 9.44 |
| 5 | 1.07 | 0,004 | 0.149 | 47.2 | 17 | 59.2 | 7.15 | 11.4 |
| 6 | 0.95 | 0,005 | 0.148 | 44.8 | 15 | 50.2 | 7.26 | 8.17 |
| 7 | 0.92 | 0,006 | 0.120 | 53.6 | 16 | 97.6 | 7.35 | 9.98 |
| 8 | 0.79 | 0,005 | 0.123 | 56.8 | 16 | 73.9 | 7.28 | 6.50 |
| 9 | 0.76 | 0,002 | 0.118 | 47.2 | 14 | 43.1 | 6.93 | 5.74 |
| 10 | 0.75 | 0,004 | 0.111 | 45.0 | 13 | 73.7 | 7.25 | 6.19 |

| A6 | | | | | | | | |
| --- | --- | --- | --- | --- | --- | --- | --- | --- |
| Week | TAN, mg/L | NH_3_‑N, mg/L | NO_2_-N, mg/L | NO_3_-N, mg/L | SO_4_, mg/L | Alkalinity, mg/L | pH | Turbidity, FNU |
| 1 | 0.59 | 0,000 | 0.194 | 41.4 |  |  |  |  |
| 2 | 0.75 | 0,004 | 0.209 | 51.0 | 15 | 67.3 | 7.43 | 7.87 |
| 3 | 0.84 | 0,000 | 0.151 | 51.8 | 19 | 72.9 | 7.77 | 6.66 |
| 4 | 0.89 | 0,005 | 0.114 | 59.0 | 22 | 78.5 | 7.32 | 6.83 |
| 5 | 1.07 | 0,005 | 0.106 | 53.8 | 22 | 73.8 | 7.31 | 11.8 |
| 6 | 0.94 | 0,007 | 0.095 | 62.2 | 25 | 71.0 | 7.39 | 7.08 |
| 7 | 0.91 | 0,005 | 0.097 | 78.6 | 32 | 61.1 | 7.29 | 6.60 |
| 8 | 1.16 | 0,005 | 0.118 | 88.8 | 36 | 73.2 | 7.27 | 8.71 |
| 9 | 0.94 | 0,002 | 0.128 | 83.8 | 36 | 30.1 | 6.86 | 8.01 |
| 10 | 0.84 | 0,005 | 0.134 | 84.4 | 33 | 66.4 | 7.27 | 5.67 |

| A7 | | | | | | | | |
| --- | --- | --- | --- | --- | --- | --- | --- | --- |
| Week | TAN, mg/L | NH_3_‑N, mg/L | NO_2_-N, mg/L | NO_3_-N, mg/L | SO_4_, mg/L | Alkalinity, mg/L | pH | Turbidity, FNU |
| 1 | 1.34 | 0,000 | 0.202 | 41.3 |  |  |  |  |
| 2 | 1.41 | 0,010 | 0.200 | 45.1 | 16 | 81.3 | 7.43 | 14.3 |
| 3 | 1.41 | 0,000 | 0.148 | 47.8 | 18 | 89.5 | 7.75 | 15.5 |
| 4 | 1.45 | 0,007 | 0.169 | 59.0 | 24 | 83.1 | 7.27 | 15.5 |
| 5 | 1.43 | 0,008 | 0.156 | 56.4 | 26 | 84.0 | 7.32 | 22.4 |
| 6 | 1.07 | 0,010 | 0.102 | 62.0 | 26 | 67.8 | 7.38 | 8.85 |
| 7 | 1.18 | 0,005 | 0.122 | 77.2 | 35 | 82.2 | 7.24 | 9.98 |
| 8 | 1.24 | 0,010 | 0.152 | 89.2 | 35 | 104.0 | 7.47 | 9.69 |
| 9 | 1.06 | 0,006 | 0.169 | 83.7 | 34 | 78.6 | 7.23 | 9.13 |
| 10 | 1.01 | 0,005 | 0.183 | 84.8 | 34 | 64.0 | 7.20 | 8.27 |

| A8 | | | | | | | | |
| --- | --- | --- | --- | --- | --- | --- | --- | --- |
| Week | TAN, mg/L | NH_3_‑N, mg/L | NO_2_-N, mg/L | NO_3_-N, mg/L | SO_4_, mg/L | Alkalinity, mg/L | pH | Turbidity, FNU |
| 1 | 0.59 | 0,000 | 0.295 | 38.6 |  |  |  |  |
| 2 | 0.67 | 0,003 | 0.304 | 48.8 | 16 | 52.0 | 7.23 | 2.41 |
| 3 | 0.67 | 0,000 | 0.215 | 46.6 | 14 | 58.8 | 7.59 | 3.13 |
| 4 | 0.79 | 0,003 | 0.176 | 53.2 | 18 | 53.3 | 7.16 | 6.61 |
| 5 | 0.99 | 0,003 | 0.141 | 50.2 | 20 | 53.0 | 7.19 | 10.0 |
| 6 | 0.66 | 0,006 | 0.113 | 49.8 | 16 | 52.3 | 7.33 | 5.15 |
| 7 | 0.82 | 0,002 | 0.147 | 56.6 | 18 | 44.1 | 7.04 | 5.16 |
| 8 | 0.70 | 0,005 | 0.151 | 57.6 | 18 | 55.6 | 7.35 | 4.50 |
| 9 | 0.79 | 0,003 | 0.218 | 54.6 | 16 | 48.5 | 7.13 | 5.11 |
| 10 | 0.74 | 0,003 | 0.267 | 49.6 | 15 | 39.6 | 7.10 | 5.57 |

Table S2. Level of detection (LOD), level of quantification (LOQ), and linearity (R^2^) of standard solutions (1‑100 mg L^-1^) used in the IC analysis.

| **Anion** | **LOD** | **LOQ** | **Linearity, R^2^** |
| --- | --- | --- | --- |
| Cl^‑^, mg L^-1^ | 0.093 | 0.176 | 0.9996 |
| NO_2_^‑^, mg L^-1^ | 0.365 | 0.476 | 0.9973 |
| NO_3_^‑^, mg L^-1^ | 0.219 | 0.243 | 0.9980 |
| SO_4_^2‑^, mg L^-1^ | 1.042 | 1.157 | 0.9988 |
| PO_4_^3‑^, mg L^-1^ | 0.097 | 0.102 | 0.9991 |

Table S3. Interday (n=5) and intraday (n=5) precision (RSD, %) of high (100 mg L^-1^) and low (10 mg L^-1^) concentrations of standard solutions used in the IC analysis.

|  | **RSD (%) Intraday** | **RSD (%) Interday** | | **RSD (%) Intraday** | **RSD (%) Interday** | |
| --- | --- | --- | --- | --- | --- | --- |
| **Anion** | **Low concentration** | | **High concentration** | | |  |
| Cl^‑^, mg L^-1^ | 0.7 | 0.7 | | 0.3 | 1.0 | |
| NO_2_^‑^, mg L^-1^ | 0.3 | 1.1 | | 0.5 | 1.2 | |
| NO_3_^‑^, mg L^-1^ | 3.3 | 3.4 | | 2.5 | 2.9 | |
| SO_4_^2‑^, mg L^-1^ | 1.0 | 4.9 | | 1.1 | 1.6 | |
| PO_4_^3‑^, mg L^-1^ | 1.0 | 2.8 | | 0.4 | 0.5 | |

Table S4. Method accuracy: spiked concentrations of selected anions (mg L^-1^) in circulating water matrix, and their recoveries (%)in the IC analysis.

| **Anion** | **Spiked concentration, mg L^-1^** | **Recovery, %** |
| --- | --- | --- |
| Cl^‑^, mg L^-1^ | 6.1‑24.6 | 99.5‑100.7 |
| NO_2_^‑^, mg L^-1^ | 7.2‑28.9 | 97.5‑104.8 |
| NO_3_^‑^, mg L^-1^ | 8.6‑34.4 | 94.8‑104.7 |
| SO_4_^2‑^, mg L^-1^ | 20.9‑83.6 | 100.9‑103.3 |
| PO_4_^3‑^, mg L^-1^ | 9.3‑37.2 | 100.4‑102.4 |

Table S5. Level of detection (LOD), level of quantification (LOQ), and linearity (R^2^) in the ICP‑MS analysis, listed for aluminium (Al^27^), cadmium (Cd^111^), cobalt (Co^59^), copper (Cu^63^), manganese (Mn^55^), nickel (Ni^60^), and lead (Pb^206^), µg L^-1^.

| **Analyte** | **LOD (µg L^-1^)** | **LOQ (µg L^-1^)** | **R^2^** |
| --- | --- | --- | --- |
| Al^27^ | 0.15 | 2.8 | 0.9979 |
| Cd^111^ | 0.12 | 0.40 | 0.9999 |
| Co^59^ | 0.27 | 0.92 | 0.9997 |
| Cu^63^ | 0.02 | 0.07 | 0.9999 |
| Mn^55^ | 0.03 | 0.11 | 0.9999 |
| Ni^60^ | 0.15 | 0.50 | 0.9999 |
| Pb^206^ | 0.06 | 0.22 | 0.9999 |

Table S6. Level of detection (LOD), quantification (LOQ), and linearity (R^2^) in the ICP‑OES analysis, listed for calcium (Ca), potassium (K), magnesium (Mg), phosphorous (P), and sulfur (S), mg L^-1^.

| **Analyte (nm)** | **LOD (mg L^-1^)** | **LOQ (mg L^-1^)** | **R^2^** |
| --- | --- | --- | --- |
| Ca (315.887) | 0.29 | 1.3 | 0.9999 |
| K (766.490) | 1.6 | 7.2 | 0.9993 |
| Mg (279.077) | 0.59 | 2.5 | 0.9999 |
| P (177.50) | 2.2 | 8.5 | 0.9991 |
| S (182.563) | 0.80 | 2.8 | 0.9999 |

Table S7. Luminescent bacteria test based on standard ISO 11348-3.2007 (inhibition, %) for the inlet water from Lake Peurunka, systems with a small side-loop (A1, A2), a large side-loop (A6, A7), and controls (A3, A8).

| **Sample** | **Inhibition, %** |
| --- | --- |
| Inlet water | 2.6 |
| Inlet water | -1.2 |
| A1 | -11.9 |
| A1 | -12.9 |
| A2 | -9.7 |
| A2 | -10.0 |
| A3 | -13.4 |
| A3 | -14.2 |
| A6 | -13.2 |
| A6 | -12.8 |
| A7 | -5.6 |
| A7 | -4.8 |
| A8 | -3.5 |
| A8 | -4.3 |

Table S8. Acute toxicity test modified from the standard ISO 6341 (immobility, %) for the inlet water from Lake Peurunka, systems with a small side-loop (A1, A2), a large side-loop (A6, A7), and controls (A3, A8).

| **Sample** | **Immobility, %** |
| --- | --- |
| Test control | 40 |
| Inlet water | 4 |
| A1 | 0 |
| A2 | 84 |
| A3 | 81 |
| A6 | 28 |
| A7 | 100 |
| A8 | 64 |


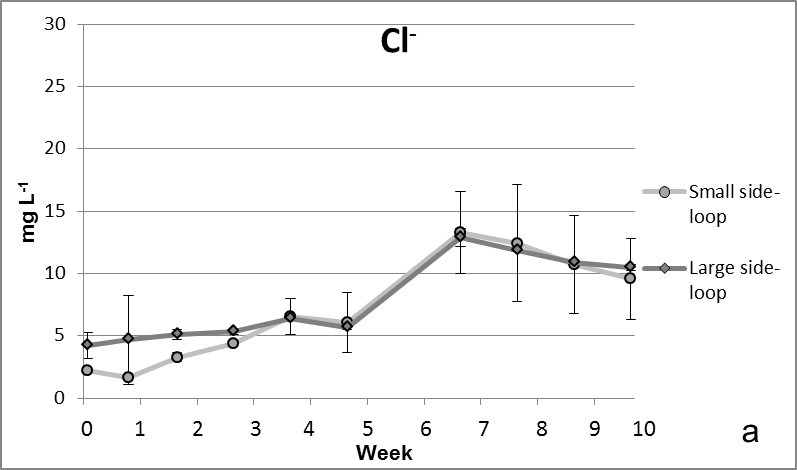

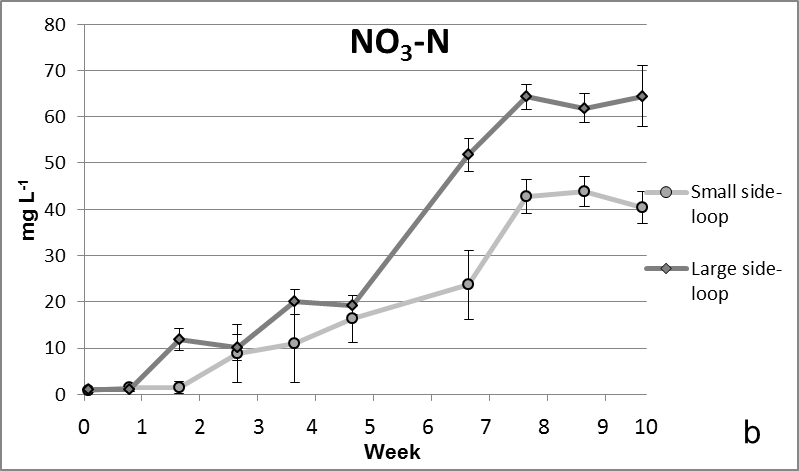


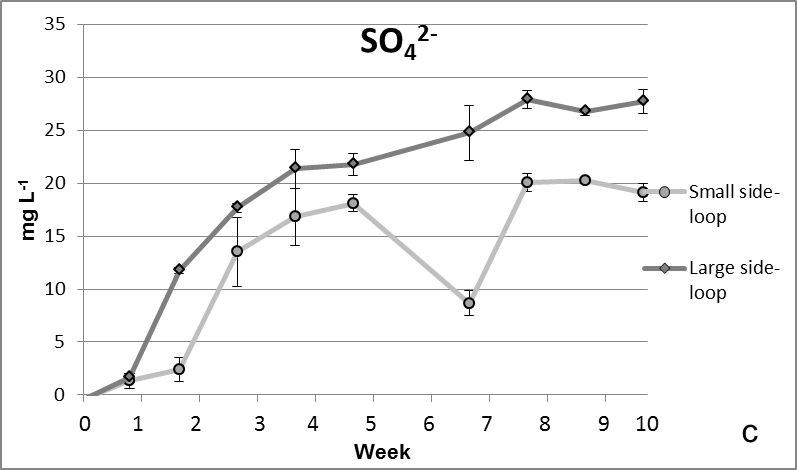

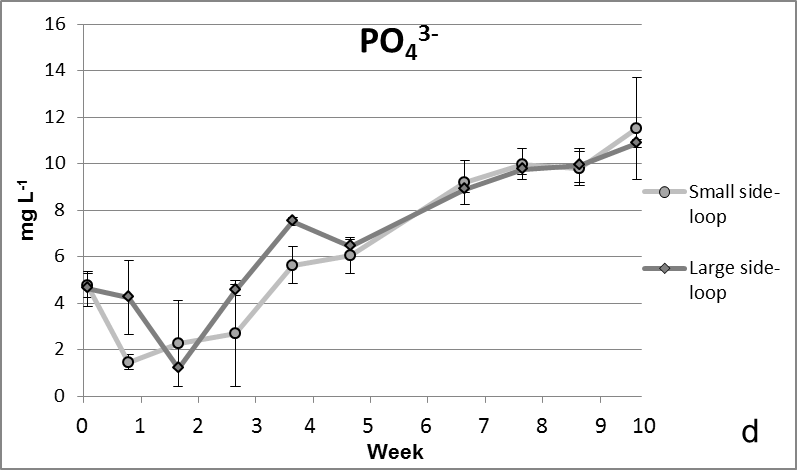


Fig. S1. Concentrations of chloride (Cl^-^, a), nitrate‑N (NO_3_‑N, b), sulfate (SO_4_^2-^, c), and phosphate (PO_4_^3-^, d) (mg L^-1^, ± SD, n=4) in circulating water after the sand filter during the 10 weeks of the experiment.
